# Synergistic senolytic–regenerative therapy significantly extends healthspan and lifespan

**DOI:** 10.1186/s12967-026-08221-y

**Published:** 2026-06-08

**Authors:** Thomas E. Ichim, Nikola Markov, Gilberto Lopes, Karenjan A. Pascual, Anastasiia Evans, Robert Reznik, Vladyslav Bykoriz, Christian A. Fortunati, Boris Minev, Roman A. Ramos, Anil Bajnath, Emma Lin, Joyce Hu, Francesco M. Marincola, Armin Rath, Barbie Barrett, Andrew Jurow, Kamlesh Kumar Sankhala, David Furman, Boris N. Reznik

**Affiliations:** 1 Immorta Bio Inc., Miami, FL US; 2 Buck Institute for Research on Aging, Novato, CA USA; 3 Department of Medical Oncology, Miller School of Medicine, University of Miami, Miami, FL US; 4 Department of Radiation Oncology, Cedars Sinai, Los Angeles, CA US; 5 Department of Radiation Oncology, University of California San Diego, San Diego, CA US; 6 Institute for Human Optimization, Hanover, MD US; 7 Department of Medicine, George Washington University, Washington, DC US; 8 BioCenturium LLC, San Diego, CA US; 9 Translational and Advanced Medicine (TAM) Biosciences, Nashville, TN USA; 10 University of California, California, US; 11 Mills-Peninsula Medical Center, Burlingame, CA US; 12 Department of Medical Oncology, Cedars Sinai, Los Angeles, CA US; 13 Immorta Bio Inc, San Diego, CA US

## Abstract

**Background:**

Current barriers to achieving radical life extension include the inability to use syngeneic, youthful mesenchymal stem cells (MSCs) and the anti-regenerative effects of senescence-associated secretory phenotype (SASP) factors. We aim to overcome this by a combination approach in which senescent cell burden is reduced utilizing SenoVax™ a dendritic cell based senolytic immunotherapy combined with syngeneic pluripotent stem cell derived MSC.

**Methods:**

We induced hepatic injury and accelerated aging using two established murine models: carbon tetrachloride (CCl₄) mediated liver injury and doxorubicin induced systemic senescence. Animals were treated with control, SenoVax, pMSCs or the combination. Outcomes included biochemical and histologic indices of liver injury, circulating and tissue biomarkers of senescence (IL-11, YKL-40, IL-6, IL-23 R) and regeneration (Klotho, FGF-2, neo-VEGF, GDF-11),

**Results:**

Both CCl₄ and doxorubicin induced a robust senescent phenotype characterized by increased pro-inflammatory and pro-fibrotic mediators and downregulation of regenerative biomarkers. Combined senolytic and pMSC therapy outperformed mono therapies and produced clear synergistic benefits, including significant biochemical improvement of liver failure parameters, reversal of accelerated aging features, and restoration of regenerative signaling pathways. Senolytic monotherapy yielded partial improvements, while pMSCs alone showed limited activity in the presence of a high senescent-cell burden.

**Conclusions:**

These findings support a mechanistic model in which senescent cells and SASP factors directly suppress MSC-mediated tissue repair. Targeted senolytic immunotherapy enhances the efficacy of regenerative interventions and represents a promising combinatorial strategy for chronic disease management and potentially for modifying biological aging itself.

**Clinical trial number:**

Not applicable.

**Supplementary Information:**

The online version contains supplementary material available at 10.1186/s12967-026-08221-y.

## Introduction

Regenerative medicine, particularly through stem cell-based therapies, holds immense potential for treating chronic diseases and mitigating the effects of aging by restoring tissue function and homeostasis. Mesenchymal stem cells (MSCs), have been extensively investigated for their paracrine effects, immunomodulatory properties, and capacity to promote tissue repair via secretion of growth factors such as vascular endothelial growth factor (VEGF) [1], and growth differentiation factor-11 (GDF-11) [2], alongside anti-aging factors like Klotho [3]. Personalized MSC (pMSC™) are a type of autologous stem cells developed by Immorta Bio which can be produced in an “age-specific” manner by controlling the extent of differentiation during generation from pluripotent stem cells [4]. MSC are attractive from an anti-aging perspective because of the studies showing young MSC can suppress and in some cases even inhibit characteristics of aging [5].

Despite promising preclinical outcomes, and one FDA approval for an orphan disease [6], clinical translation of MSC therapeutics remains limited, with many trials demonstrating modest efficacy in conditions characterized by fibrosis, inflammation, and organ failure [7]. A key barrier to successful regeneration is the accumulation of senescent cells, a hallmark of aging and chronic pathology that actively impedes stem cell function [8].

Senescent cells, induced by stressors such as oxidative damage, telomere attrition, or chemotherapeutic agents, enter a state of irreversible cell cycle arrest and secrete a constellation of pro-inflammatory cytokines, chemokines, and matrix-degrading proteins collectively known as the senescence-associated secretory phenotype (SASP) [9–11]. Prominent SASP components, include interleukin-6 (IL-6) [12–14], IL-11 [15, 16], YKL-40 (also known as chitinase-3-like-1) [17], and IL-23 receptor signaling [18]. They not only perpetuate local inflammation but also directly antagonize regenerative processes by inhibiting stem cell proliferation, differentiation, and survival [8].

For instance, elevated IL-6 has been shown to suppress MSC-mediated repair in models of liver injury [19], and doxorubicin-induced cardiotoxicity [20], while biomarkers of reduced regeneration, such as diminished Klotho, VEGF, and GDF-11, correlate with senescent burden [21]. Experimental models of organ failure and accelerated aging, including carbon tetrachloride (CCl4)-induced hepatotoxicity and doxorubicin chemotherapy, reliably recapitulate this senescent environment, manifesting as increased aging markers (e.g., IL-11, YKL-40, IL-23 r) and impaired physical capacity alongside biochemical evidence of tissue damage [22–25]. Critically, administration of isolated SASP factors like IL-6 can mimic these anti-regenerative effects, underscoring a causal role for senescent secretions in blunting stem cell efficacy [26, 27]. Emerging strategies to counteract this involve senolytic agents that selectively eliminate senescent cells or immunotherapies targeting SASP components, which have shown promise in enhancing endogenous repair and extending healthspan in preclinical studies [28]. Here, we investigate the hypothesis that senescent cells and their SASP directly impair pMSC-mediated regeneration in models of liver failure and accelerated aging. Using SenoVax™, a novel senolytic immunotherapy [29], in combination with pMSCs, we evaluate synergistic effects on biochemical markers of liver function, aging and regenerative biomarkers (IL-11, YKL-40, IL-23 r, Klotho, VEGF, GDF-11), survival and physical fitness attributes of aging. These findings support the concept that clearance of senescent cells can act as a critical adjuvant to regenerative therapies for chronic disease and aging.

## Materials and methods

### Animals

All animal experiments were conducted in accordance with the Guide for the Care and Use of Laboratory Animals (National Institutes of Health) and approved by the Institutional Animal Care and Use Committee (IACUC) at Biocentrium LLC. Male and female C57BL/6J mice (aged 8–12 weeks at the start of experiments, unless otherwise specified for aging studies) were obtained from Jackson Laboratory (Bar Harbor, ME, USA). Mice were housed in a specific pathogen-free facility under a 12-hour light/dark cycle with ad libitum access to standard chow (Purina LabDiet 5001) and water. Group sizes were determined based on power calculations assuming a 20–30% effect size, with *n* = 10 mice per group.

### Reagents and materials

Carbon tetrachloride (CCl4; ≥99.5% purity) was purchased from Sigma-Aldrich (St. Louis, MO, USA). Chemotherapy agents included doxorubicin hydrochloride (Sigma-Aldrich) administered at 2 mg/kg body weight for accelerated aging induction, based on established protocols [1]. SenoVax, a proprietary senolytic vaccine formulation targeting senescent cell surface antigens (e.g., comprising adjuvanted peptides derived from senescence-associated proteins), was provided by Immorta Bio Inc and administered subcutaneously at a dose of 100 μg per mouse in phosphate-buffered saline (PBS). Personalized mesenchymal stem cells (pMSCs) were generated by Immorta Bio as previously described [4] and characterized for surface markers (CD73+, CD90+, CD105+, CD34-, CD45-) by flow cytometry (BD FACSCalibur; BD Biosciences, San Jose, CA, USA), and cryopreserved at passage 3–5. For administration, pMSCs were thawed and resuspended in PBS at 1 × 10^6 cells per 200 μL dose and administered intravenously. ELISA kits for biomarkers (IL-11, YKL-40/CHI3L1, IL-23 R, Klotho, VEGF, and GDF-11) were obtained from R&D Systems (Minneapolis, MN, USA). Aspartate aminotransferase (AST) and alanine aminotransferase (ALT) assay kits were from Abcam (Cambridge, UK). All other reagents were of analytical grade.

### Statistical analysis

Statistical data analysis and visualization were performed in R with the following pacakges: “tidyverse”, “rstatix”, “ggpubr”, “knitr”, “broom”, “effsize”). Analysis code was written in ‘human in the loop” mode with Claude Sonnet 4.5 and every single line was reviewed. All biomarkers were analyzed with the same pipeline. First, we plotted the histograms of the mean biomarker levels with standard error (SE) bars provided as supplementary figure panel A. To avoid clutter the outcome of statistical analysis is represented in the form of heatmap as panel B. The analysis included pairwise comparisons between treatments using t-test and Bonferroni correction for multiple comparisons (Statistical significance is color coded). Our experiments follow from either CCL4 or doxorubicin induced state that we consider to be our control or reference pathological state therefore for the main figures we calculate the average level of expression of our biomarkers in this control condition and express each therapy (mono SenoVax, mono pMSC) or (combined SenoVax+pMSC) as a fold change relative to control, visualized in the main figures. For each treatment at each timepoint we also calculated an effect size based on Cohen’s method $d = \left( {{\mu _1} - {\mu _2}} \right)/S{D_{pooled}}$ where $S{D_{pooled}} = \surd \left[ {{{SD_1^2 + SD_2^2} \over 2}} \right]$. The accompanying table for each figure of the main text represents for each biomarker the average effect size (across day 7, 14 and 21) calculated for each treatment and the maximum effect size achieved in each treatment.

## Induction of accelerated aging phenotype

### Carbon tetrachloride (CCl4) model

**Fig. 1:**
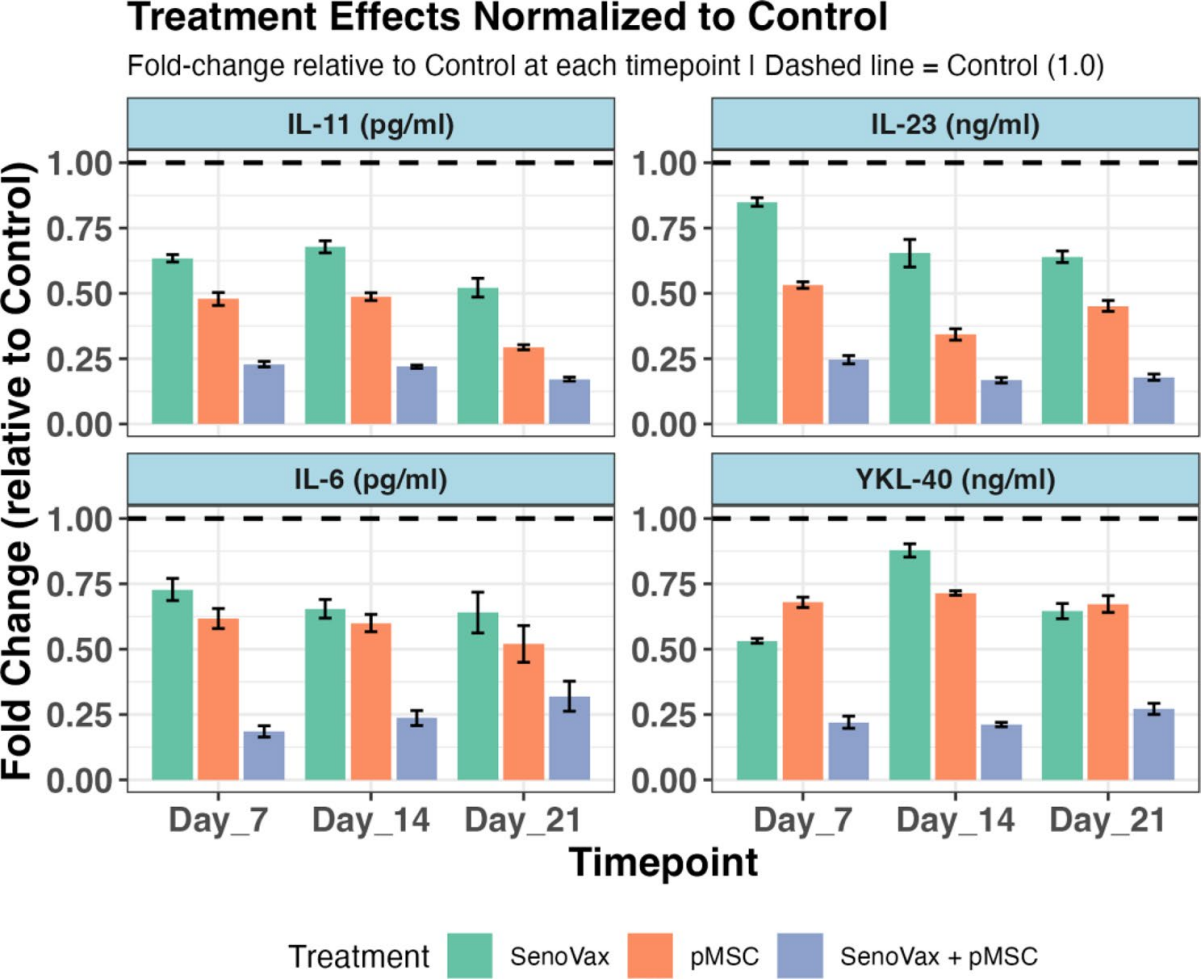
Synergistic decrease of SASP by Seno Vax and pMSC in liver failure. Liver injury was induced via carbon tetrachloride (CCl4) in 4 groups of mice prior to treatment with, Seno Vax, pMSC and combination of senovax + pMSC, a control group was untreated and served as reference. SASP related cytokines were quantified via ELISA in serum from peripheral blood. The histogram represents the mean Fold change of cytokine concentration in each treatment group with respect to the control group for the 3 timepoints. Error bars are SE. Cohen’s effect size was calculated for each treatment and is summarized in Table 1. The combined treatment showed the highest reduction in each SASP related cytokine

Accelerated aging was induced via chronic liver injury as a model of senescence-associated inflammation [2]. Mice received intraperitoneal (i.p.) injections of CCl4 (diluted 1:4 in olive oil) at 0.5 mL/kg body weight twice weekly for 8 weeks. Control mice received vehicle (olive oil) injections. Blood was collected via retro-orbital puncture under isoflurane anesthesia for biomarker analysis (Fig. 1).

### Chemotherapy model

**Fig. 2:**
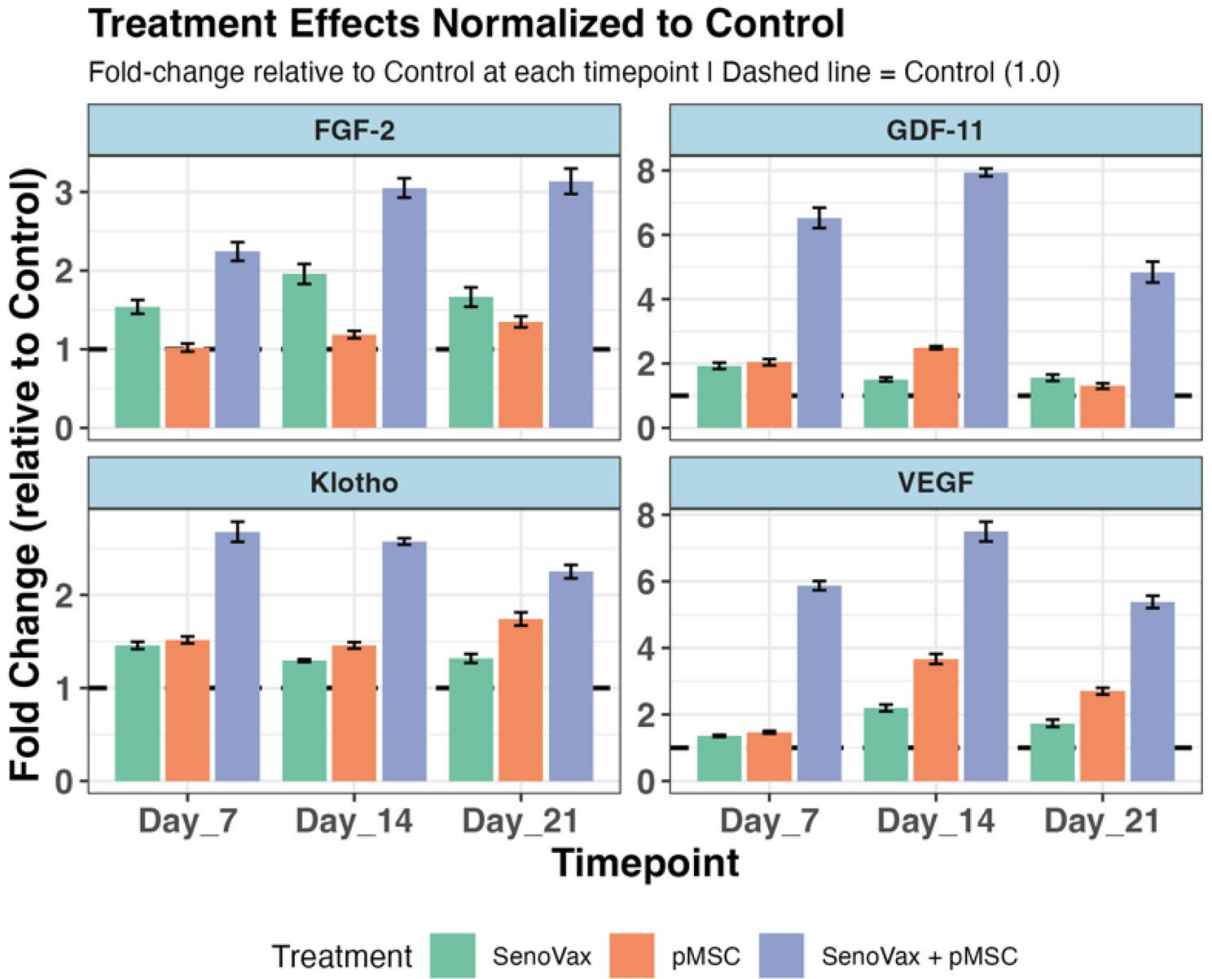
Synergistic increase of serum regenerative factors by SenoVax and pMSC in liver failure. Carbon tetrachloride was administered to induce liver injury followed by administration of SenoVax, pMSC and combination. SASP assessment was performed by ELISA. *N* = 10, error bars indicate standard deviation bars are SE. Cohen’s effect size was calculated for each treatment and is summarized in Table 2. The combined treatment showed the highest reduction in each SASP related cytokine

Chemotherapy-induced accelerated aging was modeled using repeated low-dose doxorubicin administration to mimic senescence burden without overt toxicity [3]. Mice received i.p. injections of doxorubicin (2 mg/kg) once weekly for 4 weeks. Control mice received saline injections. Blood samples were collected for biomarker assessment (Fig. 2).

### Senolytic treatment with SenoVax

**Fig. 3:**
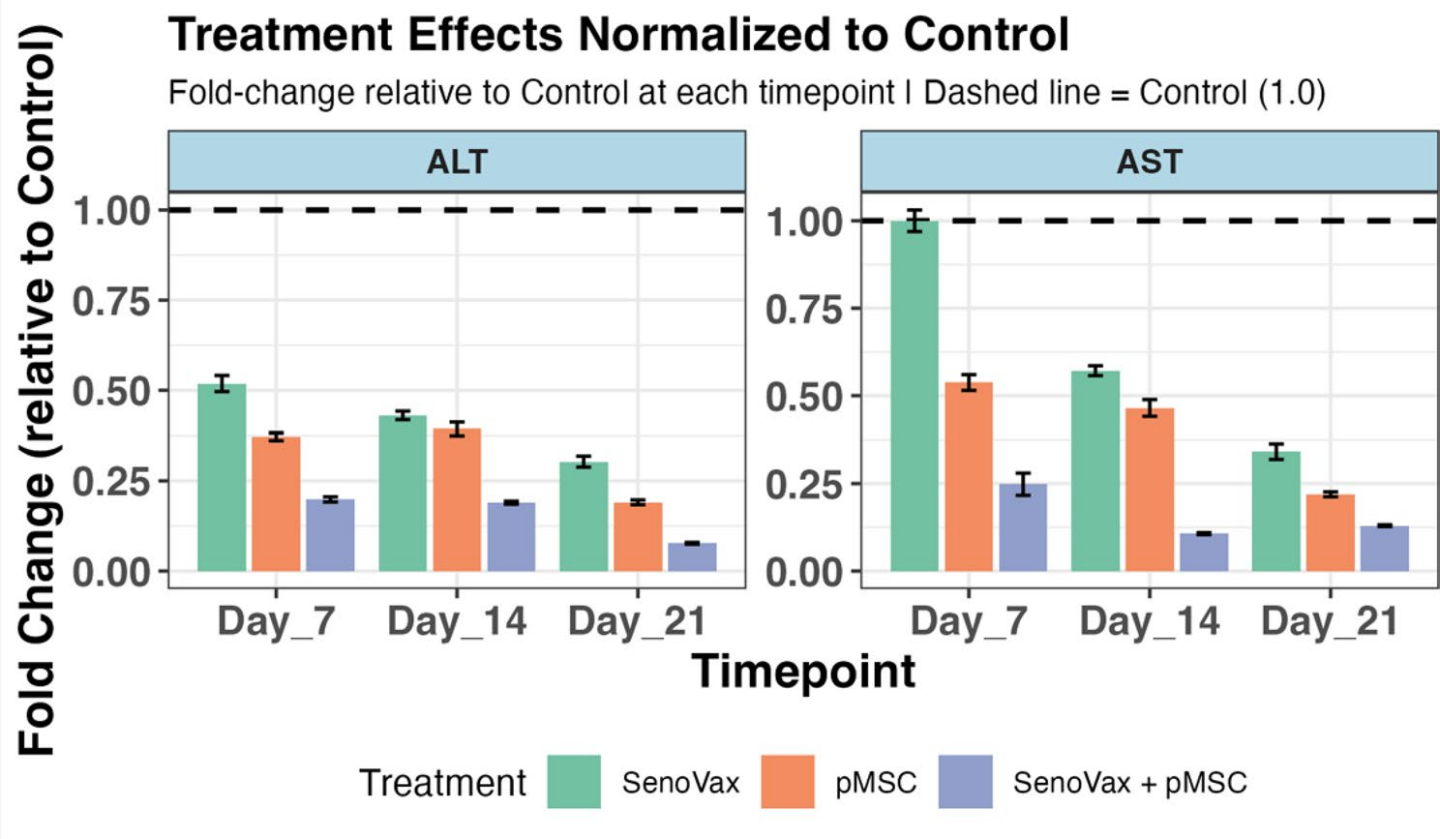
Synergistic increase of health span by SenoVax and pMSC. Liver injury was induced via carbon tetrachloride (CCl4) in 4 groups of mice prior to treatment with, Seno Vax, pMSC and combination of Senovax + pMSC, a control group was untreated and served as reference. ALT and AST were quantified via ELISA in serum from peripheral blood. The histogram represents the mean Fold change of concentration in each treatment group with respect to the control group for the 3 timepoints. Error bars are SE. Cohen’s effect size was calculated for each treatment and is summarized in Table 3. The combined treatment showed the highest reduction in each SASP related cytokine

**Fig. 4:**
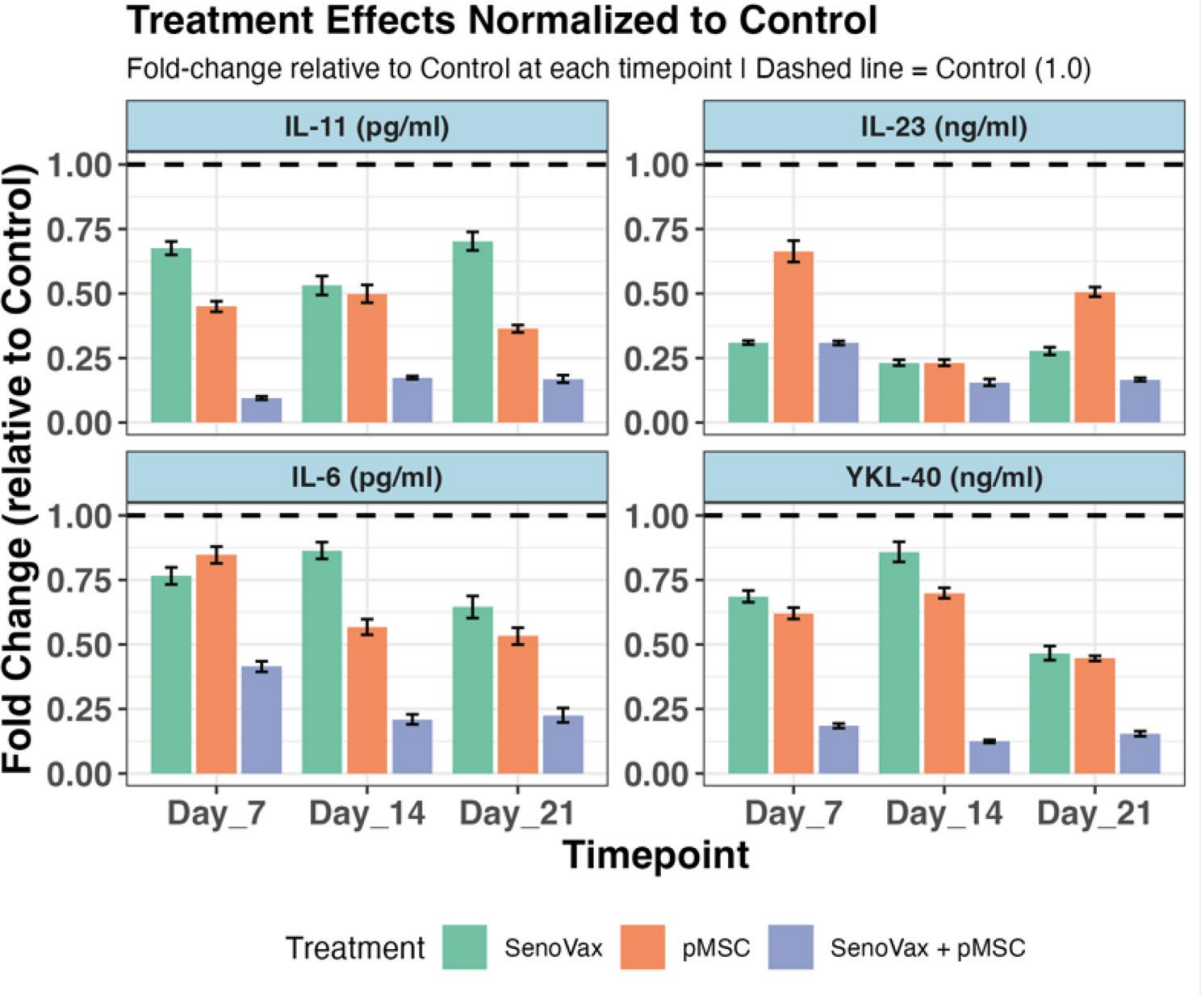
Synergistic decrease of SASP by SenoVax and pMSC in accelerated aging. Doxorubicin was administered to induce liver injury followed by administration of SenoVax, pMSC and combination. SASP assessment was performed by ELISA. The histogram represents the mean Fold change of concentration in each treatment group with respect to the control group for the 3 timepoints. Error bars are SE. Cohen’s effect size was calculated for each treatment and is summarized in Table 4. The combined treatment showed the highest reduction in each SASP related cytokine

Following aging induction, mice were treated with SenoVax to evaluate its effects on senescence-associated biomarkers. In the CCl4 model, SenoVax was administered subcutaneously starting at week 9 (post-induction) at 100 μg/mouse every 2 weeks for 4 weeks (total 3 doses). In the chemotherapy model, SenoVax was initiated at week 5 with the same dosing regimen. Blood was collected 2 weeks after the final dose for biomarker analysis (Figs. 3 and 4). Untreated induced groups served as controls.

### Combined senolytic and pMSC therapy for healthspan and lifespan enhancement

To assess synergistic effects on healthspan and lifespan, SenoVax-treated mice received additional pMSC infusions. pMSCs (1 × 10 (6) cells in 200 μL PBS) were administered intravenously via tail vein injection 1 week after the final SenoVax dose. In the CCl4 model, health span was evaluated by measuring liver function via serum AST and ALT levels at 4 weeks post-pMSC infusion (Fig. 3). In the chemotherapy model, physical healthspan was assessed at 4 weeks post-pMSC using a battery of tests: grip strength (measured with a digital force gauge; Chatillon, Largo, FL, USA; normalized to body weight), rotarod performance (accelerating from 4 to 40 rpm over 5 min; Ugo Basile, Gemonio, Italy), and open-field locomotor activity (distance traveled in 10 min; ANY-maze software, Stoelting Co., Wood Dale, IL, USA) or lifespan studies in the chemotherapy model mice were monitored until natural death or humane endpoints (e.g., >20% body weight loss, severe lethargy). Survival was tracked daily, with interventions (chemotherapy induction followed by SenoVax and pMSC as above) starting at 12 weeks of age. Untreated chemotherapy-induced and vehicle controls were included.

### Biomarker measurements

Serum was isolated from blood samples by centrifugation (1,500 × g for 10 min at 4°C) and stored at −80 °C until analysis. Biomarkers of aging and inflammation (IL-6, IL-11, YKL-40, IL-23 R) and regeneration (FGF-2, Klotho, VEGF, GDF-11) were quantified using commercial ELISA kits according to the manufacturer’s instructions. Samples were assayed in duplicate, with intra-assay coefficients of variation < 10%. Absorbance was measured at 450 nm using a microplate reader (BioTek Synergy H1, Winooski, VT, USA). Concentrations were interpolated from standard curves using a 4-parameter logistic fit.

### Liver function and physical assessments

Serum AST and ALT activities were measured spectrophotometrically using enzymatic assay kits, with results expressed in U/L. Physical performance tests were conducted in a blinded manner by trained technicians, with mice acclimated to equipment for 3 days prior to testing.

## Results

### Synergy of senolysis and stem cell administration for reduction of liver failure

In clinical situations liver failure is associated with induction of senescence [30], which is recapitulated in the murine carbon tetrachloride model [31]. Some studies have demonstrated that senolytic approaches possess activity in reduction of liver pathologies in in this model [32]. Additionally, regenerative cells such as mesenchymal stem cells (MSC) have also assisted in hepatic regeneration post injury [33]. Accordingly, we assessed the effects of senolytic immunotherapy using SenoVax, immature personalized MSC therapy (pMSC) and the combination in the animal model of CCL4 induced injury.

We observed that expression of senescence associated markers IL-11, YKL40, IL-23 receptor, and IL-6 was reduced by SenoVax and pMSC, with a larger synergistic effect observed by combination of SenoVax and pMSC (Fig. 1 and Table 1). The effects on plasma levels of markers associated with regeneration, Klotho, FGF-2, VEGF, and GDF11 (Fig. 2 and Table 2) confirmed a higher magnitude amelioration with the combination therapy (SenoVax + pMSC) compared to single therapies.

**Table 1:** Treatment effect size for decrease of SASP by SenoVax and pMSC

Biomarker	Treatment	Mean effect size	Maximal effect size
IL-11 (pg/ml)	SenoVax + pMSC	**15.1**	**21.0**
IL-11 (pg/ml)	pMSC	8.8	9.7
IL-11 (pg/ml)	SenoVax	6.3	9.4
IL-23 (ng/ml)	SenoVax + pMSC	**13.2**	**17.0**
IL-23 (ng/ml)	pMSC	8.8	12.1
IL-23 (ng/ml)	SenoVax	4.0	6.1
IL-6 (pg/ml)	SenoVax + pMSC	**7.0**	**9.1**
IL-6 (pg/ml)	pMSC	3.5	4.4
IL-6 (pg/ml)	SenoVax	2.6	3.6
YKL-40 (ng/ml)	SenoVax + pMSC	**18.5**	**30.0**
YKL-40 (ng/ml)	SenoVax	9.0	20.1
YKL-40 (ng/ml)	pMSC	7.8	12.4

**Table 2:** Treatment effect size for increase of serum regenerative factors by SenoVax and pMSC in liver failure

Biomarker	Treatment	Mean effect size	Max effect size
FGF-2	SenoVax + pMSC	8.243900179	9.87685118
FGF-2	SenoVax	3.222345567	3.6312619
FGF-2	pMSC	1.519485194	2.44697556
GDF-11	SenoVax + pMSC	27.02372011	55.3266022
GDF-11	pMSC	8.771863494	17.4169028
GDF-11	SenoVax	4.412910411	5.73388037
Klotho	SenoVax + pMSC	16.37325131	29.3006556
Klotho	SenoVax	6.606429294	10.5753441
Klotho	pMSC	6.421790955	7.33028469
VEGF	SenoVax + pMSC	19.18126657	28.0314948
VEGF	pMSC	9.930971596	16.2424886
VEGF	SenoVax	5.340312935	7.63354522

To assess the hepatic protective effect achieved by our interventions, levels of liver failure associated enzymes AST and ALT were quantified. In the control condition our therapies reliably induced elevation of the both markers of liver damage. As expected, a the largest reduction of AST and ALT was observed in the synergistic therapy (SenoVax + pMSC) with smaller effects achieved by the mono therapies alone (Fig. 3 and Table 3).

**Table 3:** Treatment effect size for increase of health span by SenoVax and pMSC

Biomarker	Treatment	Mean effect size	Max effect size
ALT	SenoVax + pMSC	17.90872678	26.06104
ALT	pMSC	11.64715805	12.18742
ALT	SenoVax	10.53615373	14.9898
AST	SenoVax + pMSC	25.44638831	40.40752
AST	pMSC	12.99519726	22.41435
ASt	SenoVax	7.765942579	1.91994

### Reduction of accelerated aging by SenoVax and pMSC

In a parallel model of therapy-induced accelerated aging, mice received low-dose doxorubicin chemotherapy for 4 weeks. Senolytics agents have previously been reported to decrease senescent phenotypes in the doxorubicin induced model [34], this model is interesting not only because it resembles accelerated aging but also because numerous cancer therapy protocols are still based on doxorubicin, protocols whose toxicity could be significantly reduced if a clinically available means of suppressing doxorubicin induced senescence was available [22, 35].

**Fig. 5:**
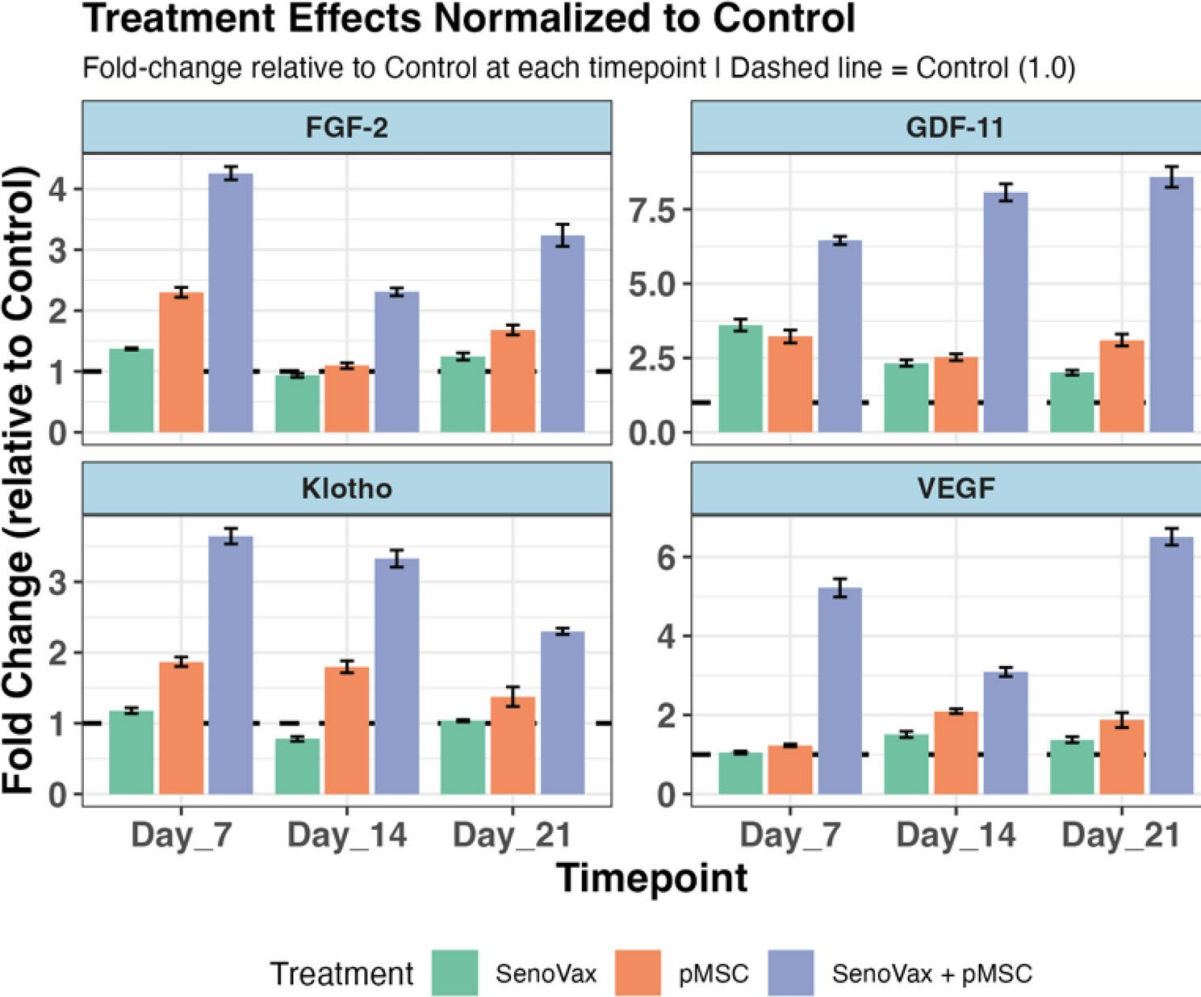
Synergistic increase in regenerative factors by SenoVax and pMSC in accelerated aging. Doxorubicin was administered to induce accelerated aging followed by administration of SenoVax, pMSC and combination., regenerative factors were quantified via ELISA in serum from peripheral blood. The histogram represents the mean Fold change of concentration in each treatment group with respect to the control group for the 3 timepoints. Error bars are SE. Cohen’s effect size was calculated for each treatment and is summarized in Table 3. The combined treatment showed the highest reduction in each SASP related cytokine

**Fig. 6:**
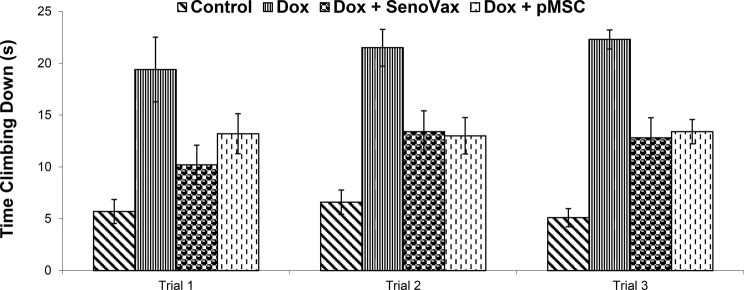
Synergistic increase in healthspan between pMSC and SenoVax. Mice were administered doxorubicin (5 mg/kg three times per week) staring day 0 for one week. After one week, mice were untreated for another week. On day 14 mice were tested for ability to “climb” down the Pole. Mice were administered a) saline (control), b) doxorubicin, c) doxorubicin + SenoVax (day −7 and day 0), d) doxorubicin + pMSC (day 0), and e) doxorubicin + combination. *N* = 10

**Fig. 7:**
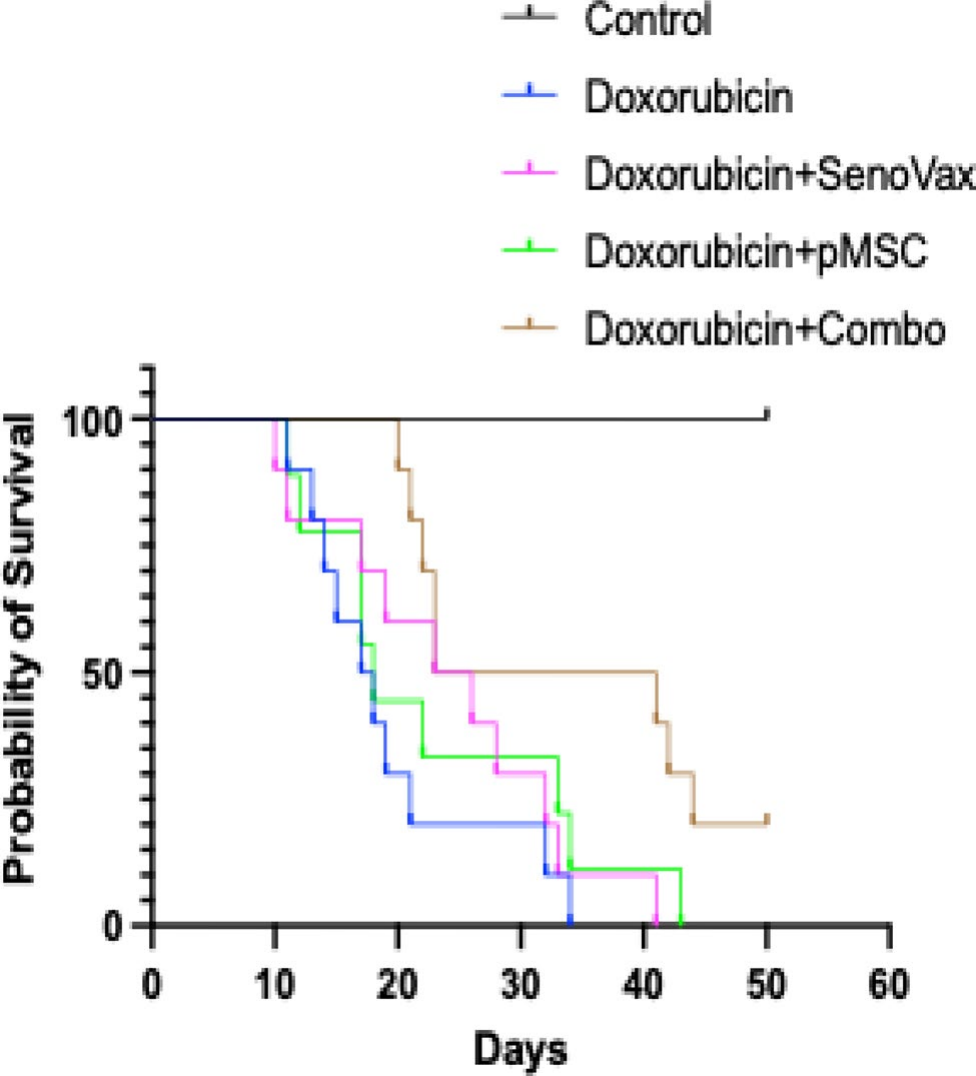
Synergistic increase in life span between pMSC and SenoVax. Mice were administered doxorubicin (5 mg/kg three times per week) staring day 0 until death. Mice were administered a) saline (control), b) doxorubicin, c) doxorubicin + SenoVax (day −7 and day 0), d) doxorubicin + pMSC (day 0), and e) doxorubicin + combination (*N* = 10 per group)

Administration of SenoVax as well as pMSC resulted in reduced expression of senescence associated biomarkers (Fig. 4 and Table 4), as well as enhanced expression of regeneration associated biomarkers (Fig. 5 and Table 5). Importantly, functional improvement as assessed by T climbing test was improved using the combination of SenoVax and pMSC (Fig. 6). Using doxorubicin as a model of accelerated aging with mortality as an endpoint, a significant synergy was observed between SenoVax and pMSC in terms of enhanced viability (Fig. 7).

**Table 4:** Treatment effect size for decrease of SASP by SenoVax and pMSC in accelerated aging

Biomarker	Treatment	Mean Effect Size	Max Effect Size
IL-11 (pg/ml)	SenoVax + pMSC	17.40119	24.95169713
IL-11 (pg/ml)	pMSC	8.100109	10.50440366
IL-11 (pg/ml)	SenoVax	4.391004	5.466725388
IL-23 (ng/ml)	SenoVax + pMSC	19.24913	27.51412291
IL-23 (ng/ml)	SenoVax	17.6448	27.74513146
IL-23 (ng/ml)	pMSC	8.062732	11.96856206
IL-6 (pg/ml)	SenoVax + pMSC	8.797795	12.14314622
IL-6 (pg/ml)	pMSC	3.924597	5.495951187
IL-6 (pg/ml)	SenoVax	2.477861	3.012474066
YKL-40 (ng/ml)	SenoVax + pMSC	16.8194	24.57565305
YKL-40 (ng/ml)	pMSC	8.99633	16.95567645
YKL-40 (ng/ml)	SenoVax	4.701808	8.126620448

**Table 5:** Treatment effect size for increase in regenerative factors by SenoVax and pMSC in accelerated aging

Biomarker	Treatment	Mean Effect Size	Max Effect Size
FGF-2	SenoVax + pMSC	11.20617846	13.92990217
FGF-2	SenoVax	4.841532146	11.4783455
FGF-2	pMSC	4.455479678	7.232190144
GDF-11	SenoVax + pMSC	23.43312955	26.36326766
GDF-11	SenoVax	7.283530588	8.841855341
GDF-11	pMSC	6.069835862	7.960234502
Klotho	SenoVax + pMSC	15.82677816	17.35642
Klotho	pMSC	4.413452291	7.126938382
Klotho	SenoVax	1.923202437	2.594675509
VEGF	SenoVax + pMSC	11.11450911	14.25154373
VEGF	pMSC	5.581979823	11.52341065
VEGF	SenoVax	2.006179952	3.13465522

## Discussion

The present study demonstrates that combined senolytic immunotherapy with SenoVax™ and regenerative therapy using personalized mesenchymal stem cells (pMSCs) synergistically ameliorates accelerated aging phenotypes in murine models of liver injury and chemotherapy-induced senescence. By targeting senescent cells and their deleterious secretions, this dual approach not only reverses inflammatory and regenerative biomarker imbalances but also enhances health span metrics such as liver function and physical performance and extends lifespan in a synergistic manner. These findings underscore the critical role of senescent cell burden as a barrier to effective stem cell-based regeneration [8], supporting the hypothesis that senolytics serve as a vital adjuvant to regenerative medicine for chronic diseases and aging.

In both the CCl4-induced liver failure and doxorubicin chemotherapy models, we observed robust induction of an accelerated aging phenotype, characterized by elevated pro-inflammatory SASP markers (IL-11, YKL-40, IL-23 R) and diminished regenerative factors (Klotho, VEGF, GDF-11). This aligns with established literature showing that stressors like chemical toxins and chemotherapeutic agents trigger cellular senescence, leading to a self-perpetuating inflammatory milieu that exacerbates tissue dysfunction [9–11, 22–25].

In some studies, it has been demonstrated that SASP proteins are not only age associated markers, but are actually involved in the pathology of age-associated diseases. For example, administration of IL-11 has been shown to exacerbate liver failure [36], and administration of agents that suppress IL-11 appears to have therapeutic effects [37]. Inflammatory effects of YKL-40 are well known, with this cytokine having been implicated in conditions such as liver failure [38], diabetes [39], macular degeneration [40], and heart failure [41, 42].

For instance, CCl4 chronic exposure models hepatic fibrosis and senescence-associated inflammation [2, 25], while low-dose doxorubicin recapitulates therapy-induced senescence without immediate lethality, mimicking clinical scenarios of cancer survivorship where accelerated aging is a major comorbidity [3, 20, 22]. The time-dependent progression of biomarker changes in our models, peaking at 8 weeks for CCl4 and 4 weeks for doxorubicin, highlights the rapid accumulation of SASP factors, which we further corroborated by noting that exogenous IL-6 administration replicates these effects [26]. This causal link emphasizes how SASP components, such as IL-6, IL-11, and YKL-40, directly antagonize regenerative processes, consistent with reports of IL-6 suppressing MSC-mediated repair in liver and cardiac injury [12–14, 19, 20].

SenoVax monotherapy effectively mitigated these senescence-driven alterations, reducing inflammatory markers by 2.0- to 2.7-fold and boosting regenerative biomarkers by 45–60% across models. This reversal suggests targeted clearance of senescent cells, likely through immune-mediated elimination of senescence-associated antigens, thereby attenuating SASP output [27]. Preclinical senolytics, including small molecules and immunotherapies, have similarly shown efficacy in clearing senescent cells and improving tissue function [15, 27], but SenoVax’s vaccine-like formulation offers potential advantages in durability and specificity, avoiding off-target effects seen with broad-spectrum agents [8]. Notably, the absence of sex differences in our biomarker responses aligns with broader observations that senescence pathways are conserved across sexes, though future studies should explore hormonal influences in aged cohorts [17]. We acknowledge limitations of the accelerated aging model used and studies are underway exploring therapeutic effects in natural aging conditions.

One of the questions remaining in the current study is the identification of specific senescence associated antigens that are being targeted by SenoVax induced immunity. Data submitted to the Food and Drug Administration as part of IND 30745 demonstrated no indication of autoimmune in chronic and acute administration studies (data not shown). We have previously reported generation of senolytic antibodies which specifically lyse senescent but not control fibroblasts [29]. The existence of senescence-associated immunogenic molecules has previously been demonstrated, for example, immunogenicity of uPAR was shown by Amor et al. who were able to generate senolytic CAR-T which possessed therapeutic effect in a variety of age-related models [32].

The synergistic benefits of combining SenoVax with pMSCs represent a key advancement, addressing a major limitation in regenerative medicine: the hostile microenvironment imposed by senescent cells [8, 26]. In the CCl4 model, combined therapy reduced AST and ALT by 58–62%, surpassing monotherapies (32–40% reductions), indicating enhanced hepatic regeneration. This synergy likely stems from SenoVax creating a permissive niche by diminishing SASP-mediated inhibition, allowing pMSCs to exert their paracrine effects more effectively promoting angiogenesis via VEGF, anti-aging via Klotho, and tissue repair via GDF-11 [1–3, 5]. Similarly, in the doxorubicin model, the combination improved physical performance in the pole test by 65%, reflecting preserved motor coordination and strength, which are hallmarks of healthspan [23, 24]. These functional gains extend prior findings where MSC-derived extracellular vesicles reduce senescence and extend healthspan in aging models [5], but our use of personalized pMSCs generated in an age-specific manner [4] may enhance autologous compatibility and efficacy.

Critically, the lifespan extension in the doxorubicin model, with 50% survival at Day 35 and 20% at Day 40 for the combination versus complete mortality by Day 30 in untreated controls, highlights the translational potential for managing chemotherapy sequelae. Monotherapies extended median survival modestly (to ~Day 35), but the synergy (*p* < 0.05 vs. monotherapies) suggests complementary mechanisms: SenoVax clears senescent debris, while pMSCs replenish regenerative signals [8, 27]. This mirrors recent reports of senolytics rejuvenating cardiomyopathy in human organoids [23] and alleviating doxorubicin cardiotoxicity [22], but our integrated approach addresses systemic aging, not just organ-specific damage.

Mechanistically, the observed reversals may involve SASP inhibition alleviating stem cell suppression. For example, IL-11 and IL-6 sustain senescence via NF-κB and cGAS-STING pathways [12, 14, 15], while YKL-40 promotes fibrosis and inflammation in myopathies and pulmonary disease [17, 18]. By reducing these, SenoVax likely enhances pMSC engraftment and secretion of rejuvenating factors like GDF-11, which counters bone loss and senescence [2, 21, 26]. The upregulation of Klotho, an anti-aging hormone, further supports systemic benefits, as its decline correlates with frailty and reduced lifespan [3, 21]. Intriguingly, our data imply that SASP factors directly impair pMSC function, as biomarker shifts mimic those in IL-6-exposed models [19, 26], reinforcing the need for senescent clearance prior to regenerative interventions. To our knowledge this is the first utilization of a combination approach involving senescent cell removal followed by administration of regenerative cells. The use of senescent cell removal to help liver failure [43], and extend lifespan has previously been reported [44], as well as the administration of regenerative cells for liver failure [45] and life span extension has been previously reported [46].

Despite these strengths, limitations warrant consideration. Our models, while robust, are acute/subchronic and may not fully capture natural aging’s complexity, where senescent accumulation is gradual [10]. Group sizes (*n* = 10) provided sufficient power for primary endpoints but may limit detection of subtle sex or age interactions. Additionally, while biomarkers like IL-23 R indicate inflammatory senescence [18], direct quantification of senescent cell clearance (e.g., via p16^INK4a or SA-β-gal staining) would strengthen mechanistic claims. Future studies should incorporate histological analyses and extend to aged or comorbid models to assess long-term durability [6, 7]. Clinically, translating SenoVax and pMSCs requires safety evaluations, particularly immunogenicity and off-target effects in humans [27], though the autologous nature of pMSCs mitigates rejection risks [4].

In conclusion, this study provides compelling evidence that senescent cells and SASP proteins hinder stem cell-mediated regeneration, and that senolytic immunotherapy like SenoVax acts as a synergistic adjuvant to pMSCs. These findings pave the way for integrated approaches to combat chronic disease and aging, emphasizing the need for niche-modifying therapies to enhance regenerative outcomes.

## Electronic supplementary material

Below is the link to the electronic supplementary material.

Supplementary material 1

Supplementary material 2

## Data Availability

All data generated and analyzed in this study are included in this article.
