# Supplementary material for: Synergistic senolytic–regenerative therapy significantly extends healthspan and lifespan
Source: J Transl Med. 2026 Jun 8;24:745. doi: 10.1186/s12967-026-08221-y (PMC13244891; doi:10.1186/s12967-026-08221-y)
Supplement: Supplementary file 1 — Supplementary material 1 [file 12967_2026_8221_MOESM1_ESM.pptx]

## Slide 1
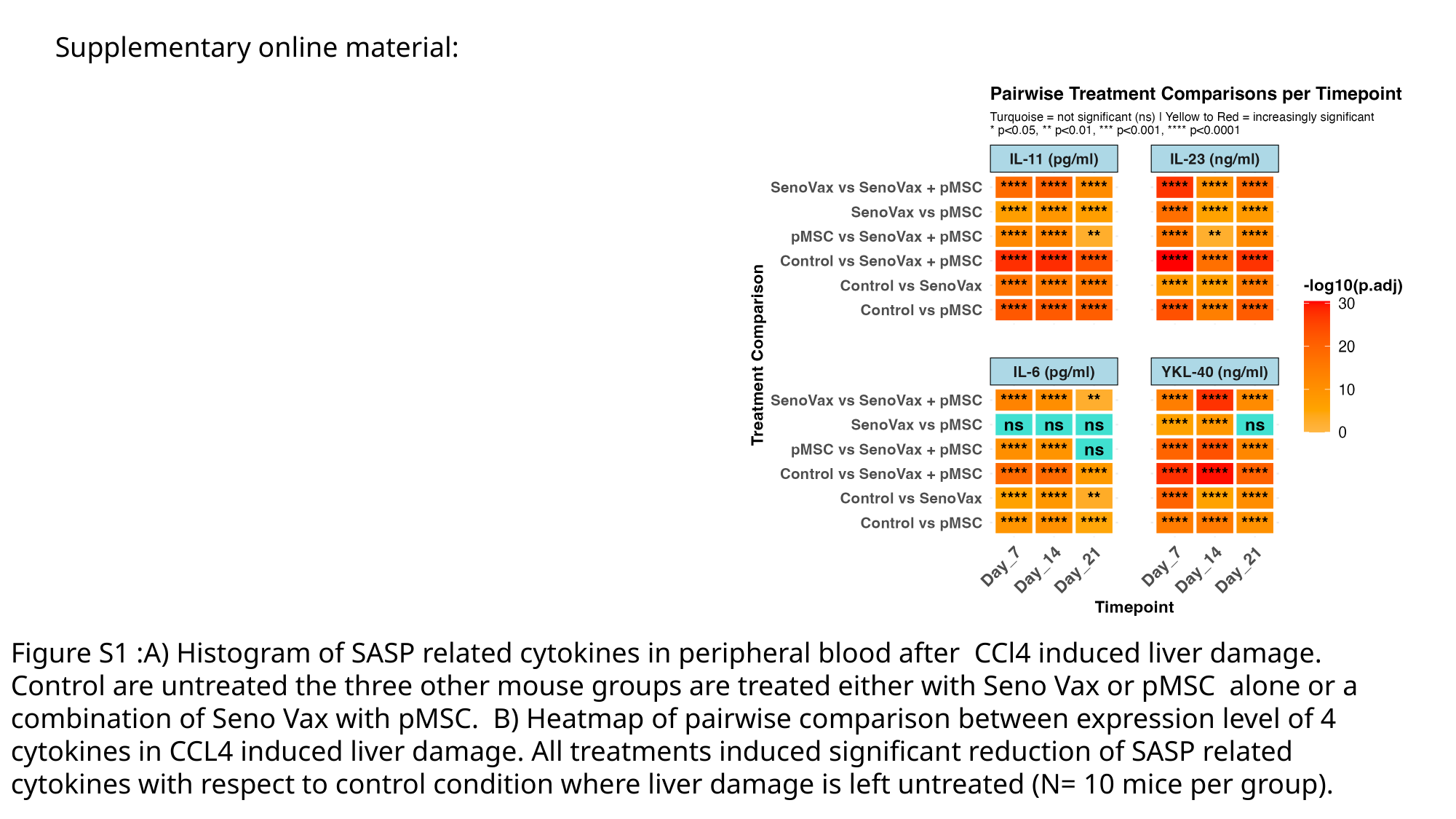

Supplementary online material:
Figure S1 :A) Histogram of SASP related cytokines in peripheral blood after CCl4 induced liver damage. Control are untreated the three other mouse groups are treated either with Seno Vax or pMSC alone or a combination of Seno Vax with pMSC. B) Heatmap of pairwise comparison between expression level of 4 cytokines in CCL4 induced liver damage. All treatments induced significant reduction of SASP related cytokines with respect to control condition where liver damage is left untreated (N= 10 mice per group).

## Slide 2
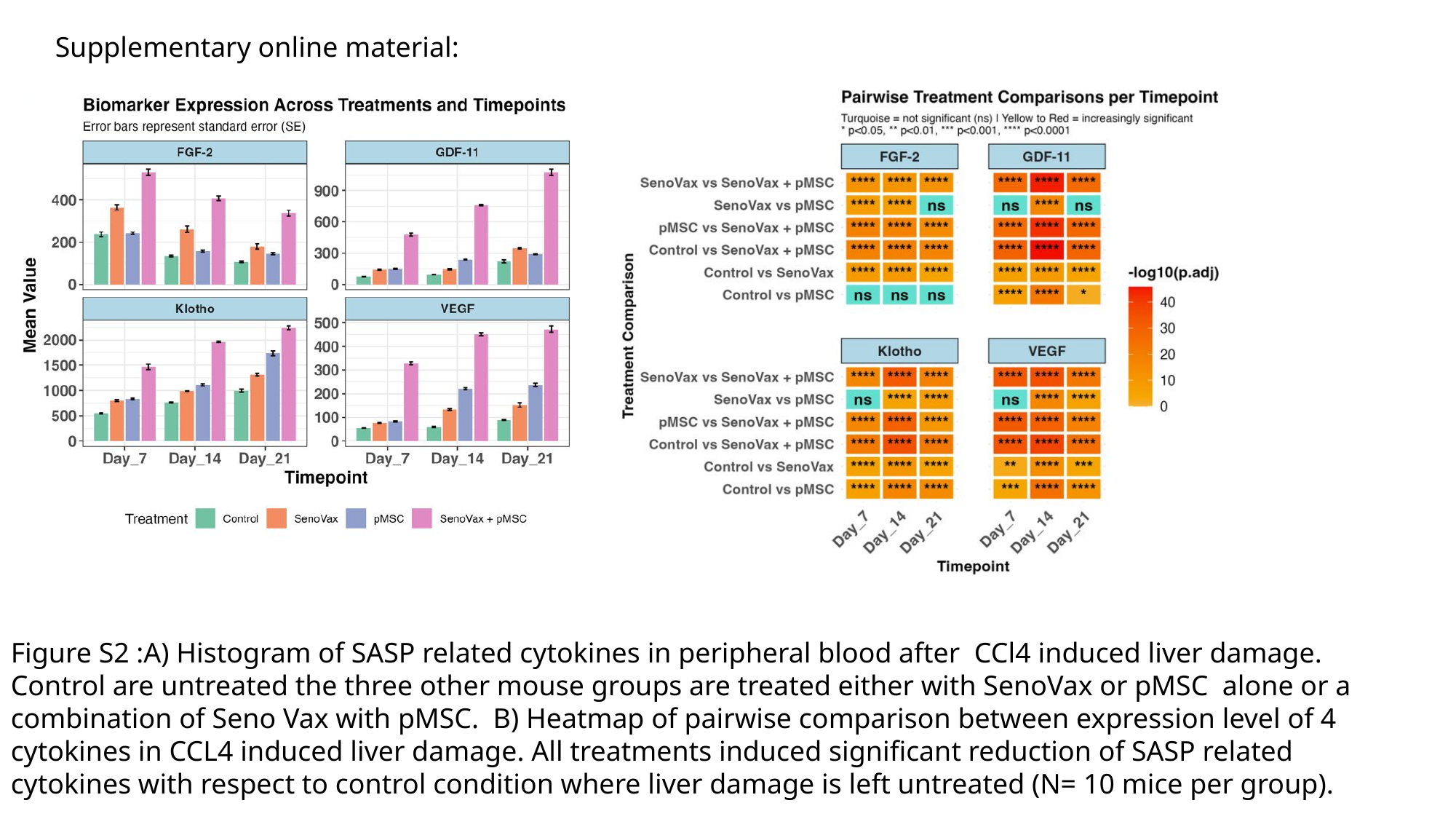

Supplementary online material:
Figure S2 :A) Histogram of SASP related cytokines in peripheral blood after CCl4 induced liver damage. Control are untreated the three other mouse groups are treated either with SenoVax or pMSC alone or a combination of Seno Vax with pMSC. B) Heatmap of pairwise comparison between expression level of 4 cytokines in CCL4 induced liver damage. All treatments induced significant reduction of SASP related cytokines with respect to control condition where liver damage is left untreated (N= 10 mice per group).

## Slide 3
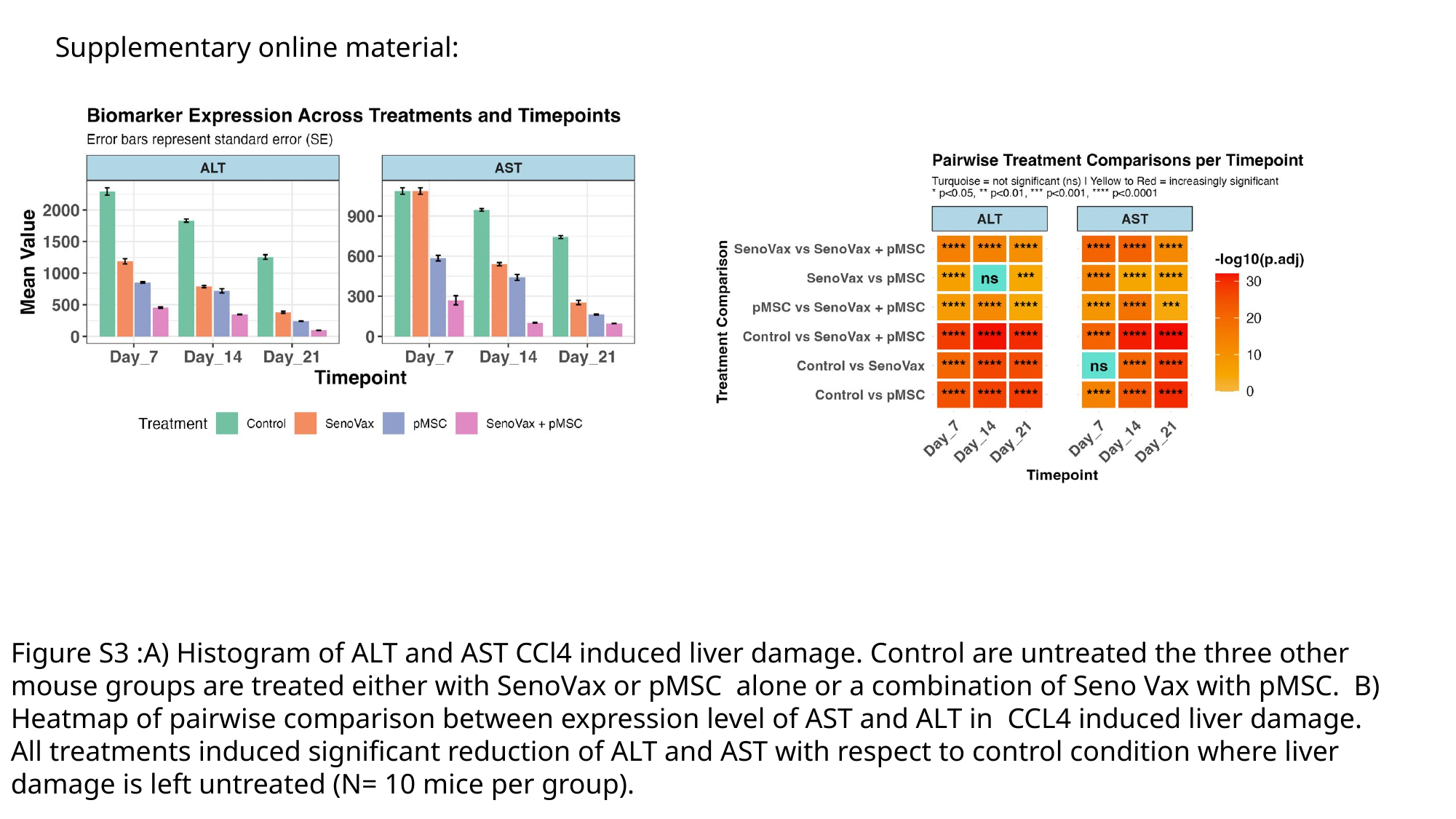

Supplementary online material:
Figure S3 :A) Histogram of ALT and AST CCl4 induced liver damage. Control are untreated the three other mouse groups are treated either with SenoVax or pMSC alone or a combination of Seno Vax with pMSC. B) Heatmap of pairwise comparison between expression level of AST and ALT in CCL4 induced liver damage. All treatments induced significant reduction of ALT and AST with respect to control condition where liver damage is left untreated (N= 10 mice per group).

## Slide 4
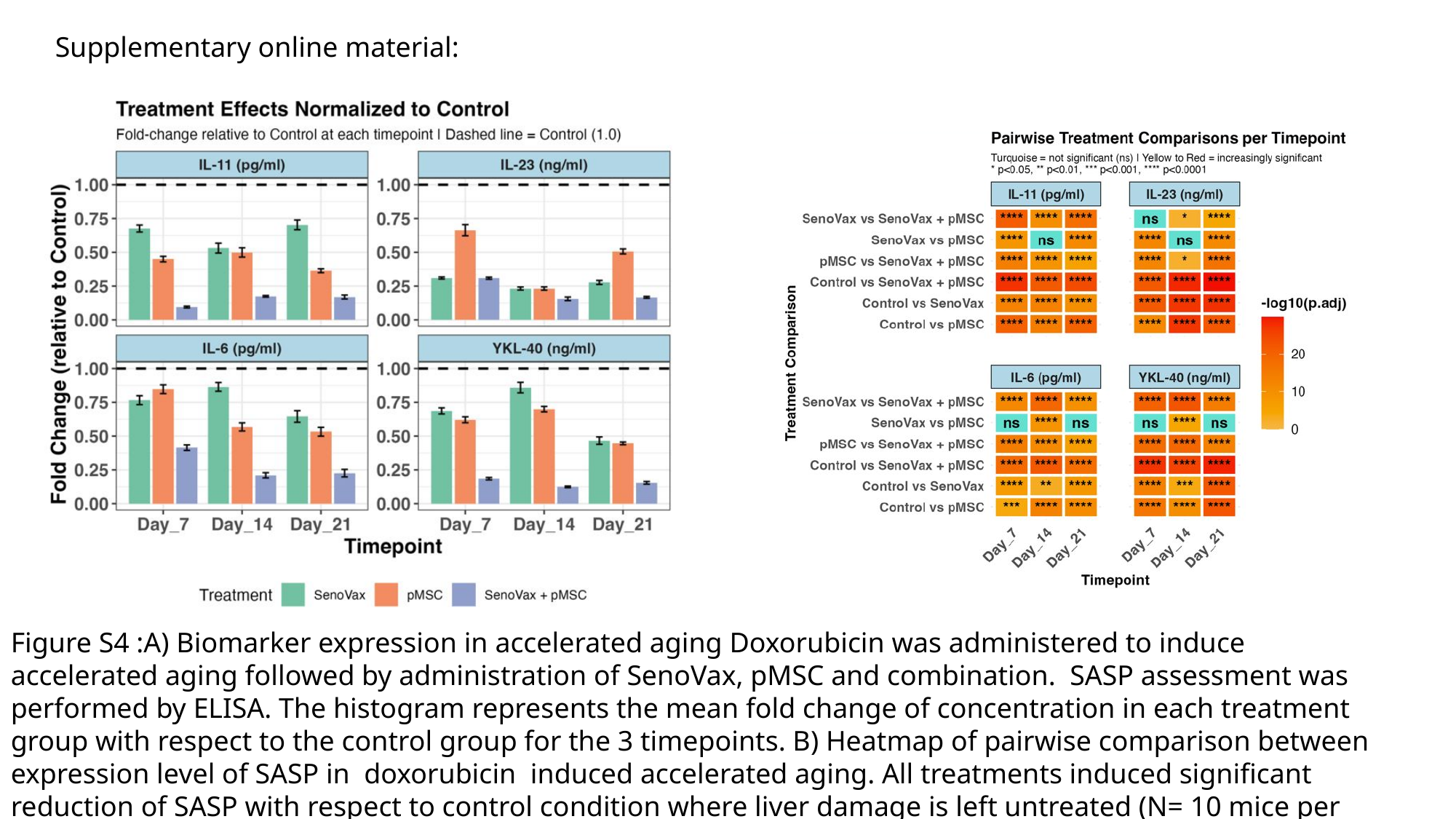

Supplementary online material:
Figure S4 :A) Biomarker expression in accelerated aging Doxorubicin was administered to induce accelerated aging followed by administration of SenoVax, pMSC and combination. SASP assessment was performed by ELISA. The histogram represents the mean fold change of concentration in each treatment group with respect to the control group for the 3 timepoints. B) Heatmap of pairwise comparison between expression level of SASP in doxorubicin induced accelerated aging. All treatments induced significant reduction of SASP with respect to control condition where liver damage is left untreated (N= 10 mice per group)..

## Slide 5
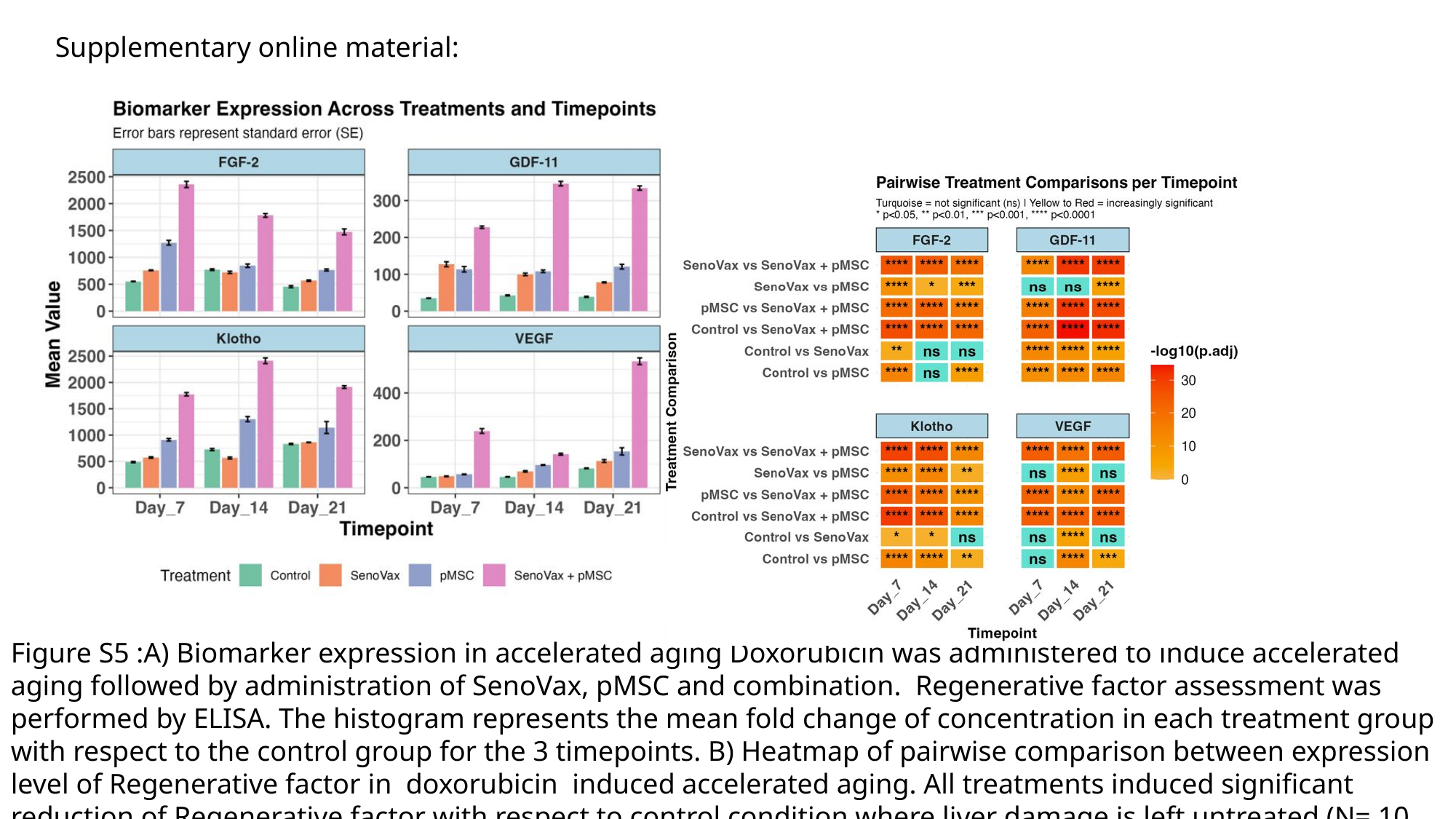

Supplementary online material:
Figure S5 :A) Biomarker expression in accelerated aging Doxorubicin was administered to induce accelerated aging followed by administration of SenoVax, pMSC and combination. Regenerative factor assessment was performed by ELISA. The histogram represents the mean fold change of concentration in each treatment group with respect to the control group for the 3 timepoints. B) Heatmap of pairwise comparison between expression level of Regenerative factor in doxorubicin induced accelerated aging. All treatments induced significant reduction of Regenerative factor with respect to control condition where liver damage is left untreated (N= 10 mice per group).
